# Evaluation of the accuracy of a low-cost external tocodynamometer in a pilot study in Malawi

**DOI:** 10.1371/journal.pone.0323972

**Published:** 2025-05-16

**Authors:** Alex Kortum, Christina Samuel, Theresa Sonka, Lisette Tanner, Benjamin J.F. Huntley, Ahmed Abouseif, Z. Maria Oden, Richard A. Schwarz, Jennifer Carns, Suneet P. Chauhan, Phylos Bonongwe, Rebecca Richards-Kortum

**Affiliations:** 1 Department of Bioengineering, Rice University, Houston, Texas, United States of America; 2 Rice360 Institute for Global Health Technologies, Houston, Texas, United States of America; 3 University of Texas Health Sciences Center at Houston, Houston, Texas, United States of America; 4 Ministry of Health, Queen Elizabeth Central Hospital, Blantyre, Malawi; Johns Hopkins University School of Medicine, UNITED STATES OF AMERICA

## Abstract

Uterine contraction monitoring during labor has been linked to improved maternal outcomes. However, performing this monitoring can be challenging for financial and logistical reasons in low resource settings. This proof-of-concept study aimed to compare the accuracy of a low cost external tocodynamometer we developed to that of a commercially available external tocodynamometer. In total, 60 patients with anticipated vaginal deliveries at a hospital in Blantyre, Malawi were enrolled. Both the research device and the commercial device were secured to the patients, and traces were recorded simultaneously from each device. Trace pairs were split into 10 minute segments, and contraction locations were independently annotated on a selection of 75 contemporaneous trace pairs from 38 out of 60 patients by two expert clinicians. In total, 484 contractions were marked on the research device, and 465 contractions were marked on the commercially available device, 312 of which were marked consistently on both devices. The average consistency of marked contractions on the same device between the two observers was 0.89 for the research device compared with 0.84 for the commercially available device. The average consistency between the two devices using a 10-patient rolling average increased from 0.50 at the beginning of the study to 0.64 at the end. The annotated traces from the two devices suggested the same clinical management 72% of the time. The research device displayed reasonable agreement with the commercially available tocodynamometer in detecting contractions. The increase in the measured consistency over the course of the study suggests that improving usability of the device to ensure better positioning on the patient could result in improved performance. Further studies assessing the accuracy and usability of the device are needed.

## Introduction

During normal labor and delivery, regular and effective contractions of the uterine myometrium result in dilation and effacement of the cervix; the frequency, duration, and strength of uterine contractions increases as labor progresses [1]. When inadequate uterine contractions cause delayed labor, augmentation of labor may be performed to stimulate more effective uterine contractions [2]. Uterine tachysystole, a condition characterized by uterine contraction frequency in excess of five contractions in 10 minutes averaged over 30 minutes, is a complication most commonly seen in augmentation of labor, and when not identified and managed early, can result in prolonged oxygen deprivation to the fetus and is associated with adverse neonatal outcomes [3].

Uterine contractions can be monitored externally through manual palpation or external tocodynamometry, or internally with an intrauterine pressure catheter (IUPC). Although internal measurement with an IUPC is the only option for characterizing the strength of uterine contractions, it is invasive in nature and increases the risk of complications. Therefore, external monitoring of contractions, which can provide information about the frequency and duration of contractions, is performed whenever possible. While low-cost options exist for continuous fetal heart rate monitoring such as the Moyo Fetal Heart Rate Monitor [4], the current standard for continuous external monitoring of uterine contractions in high resource settings, an external tocodynamometer, can be prohibitively expensive for lower resource areas, costing thousands of dollars [5]. Other monitoring devices using electrohysterography, such as the Avalon [6], Novii [7], Nemo [8], and ANNE [9], have recently become available, but may require significant additional training and infrastructure before widespread adoption could occur, particularly in low resource settings. The recommended method for monitoring contractions in low resource settings involves having a nurse periodically palpate contractions manually and record them on a partograph [10], which is logistically difficult in areas where nurse to patient ratios are low and is prone to error by nature of its subjectivity.

Contraction monitoring is of significant importance when augmentation of labor is performed, as insufficient monitoring of the progression of labor is associated with adverse outcomes for the maternal-neonatal dyad. The WHO recommends that women undergoing augmentation of labor not be left unattended, that partographs are used to monitor progression of labor, and that uterine contractions are regularly monitored [11]. However, even intermittent manual monitoring of uterine contractions and other parameters associated with the progression of labor cannot be accomplished with the desired frequency in settings where nurse to patient ratios can approach 1:15 [12–14].

This paper describes a low cost, reusable external tocodynamometer, termed Optoco, which we developed for continuous monitoring of uterine contractions in low resource settings. We present results of a proof-of-concept study designed to compare the accuracy of Optoco to that of an existing commercial tocodynamometer.

## Materials and methods

### Instrument design

**Fig 1:**
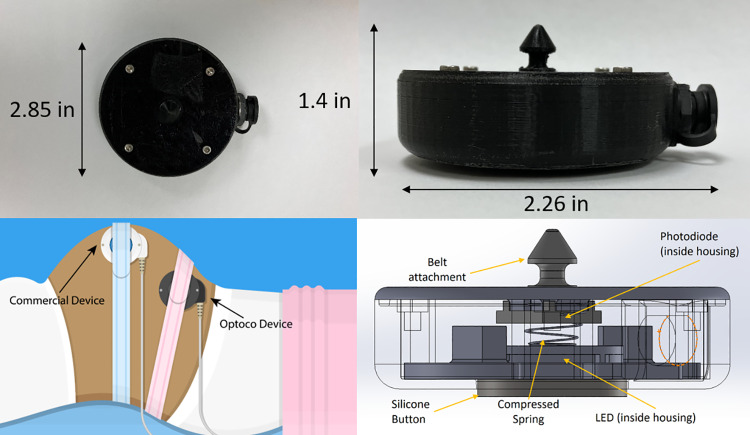
Optoco schematic. (Top Left and Top Right) Photos of Optoco device with dimensions. (Bottom Left) Drawing of the Optoco device and commercial device as they would be secured to a patient during data acquisition. (Bottom Right) Schematic diagram of Optoco components.

As shown in Fig 1, Optoco consists of an LED and photodiode enclosed in a sealed 3D printed housing. The housing is attached to a flexible silicone button that protrudes from the bottom of the device and is placed in contact with the patient’s abdomen. The photodiode is fixed at the top of the sensor; the LED and photodiode are connected by a compressible spring. When a contraction occurs, the spring is compressed, pushing the LED closer to the photodiode and creating a detectable change in signal from the photodiode. As the contraction subsides, the spring pushes the button back to its rest position.

A transimpedance amplification circuit and Arduino microcontroller connected to the device via a cable are used to continuously read the signal from the photodiode during monitoring. The brightness of the LED was adjusted to provide the maximum difference in photodiode signal between when the button was fully depressed and fully released. The total cost of the device, including the electronics used to read the signal, is approximately $56.

During operation, the device is secured to the abdomen of the patient with an elastic band using a button at the top of the device. The Arduino is connected to a laptop, and the readout of the photodiode subjected to a moving average filter is displayed to the user, producing a uterine contraction trace similar to that of a tocodynamometer.

### Laboratory calibration and testing

A GE Corometrics 174 maternal/fetal monitor connected to a Nautilus Toco Transducer was used as the gold standard reference for uterine activity. During operation, the GE uterine activity monitor was secured to the abdomen of the patient using either the same elastic band used to secure Optoco, or a second elastic band, at the healthcare provider’s discretion. A second laptop was connected directly to the monitor and digital data were collected via the monitor’s Model 115 compatible communications protocol. Results showing contraction strength vs time were displayed to the clinician using a program written in MATLAB as described below.

### Study information

A study was conducted at Queen Elizabeth Central Hospital (Blantyre, Malawi) to compare the performance of Optoco against that of the commercially available gold standard external tocodynamometer. The study protocol was approved by the National Health Sciences Research Committee in Malawi (19/11/2444) and the Institutional Review Board at Rice University (FY2020–190) prior to study initiation. Written informed consent was obtained from participants before they were enrolled in the study. Additional information regarding the ethical, cultural, and scientific considerations specific to inclusivity in global research is included in the Supporting Information (S1 Checklist). Women were approached for the study if they were 18 or older with an anticipated vaginal delivery and were willing and able to provide informed consent.

Women who consented to the study had both the Optoco device and the commercial external tocodynamometer (GE Corometrics Nautilus Toco Transducer) placed and secured on their abdomen, and data from both devices were collected simultaneously. The study nurses were asked to periodically observe uterine contraction traces from both devices and adjust the placement of either device if the signal was low, but it was acknowledged that the optimal placement of either device could impact the placement, and thus the accuracy, of the second device. There were no restrictions placed on the patients’ movement beyond those in place as part of the standard of care. A total of 60 patients were enrolled between March 25, 2021 and October 22, 2021.

### Analysis

S1 Fig gives an overview of how the data were processed. The first and last five minutes of all traces were excluded to remove motion artifacts created by placing or removing the sensors. Because the two laptops were not connected to the internet due to lack of internet access at the site, their clocks were not synchronized during the course of the study. To account for any difference in timestamps generated by the two devices, the traces were aligned according to the following procedure: the two traces were resampled to the same data rate of 8 Hz, and the first ten minutes of the Optoco trace were selected. The commercial toco trace and ten-minute Optoco segment were then normalized, and the cross correlation of the two vectors was computed, with a limit of two minutes in either direction. The Optoco segment was then advanced by one minute (from 0–10 minutes to 1–11 minutes), and the cross correlation was computed again. This was repeated until the end of the trace had been reached. The median offset suggested by the cross correlation values was then used for the entire trace.

After alignment, the two traces were split into corresponding 10 minute segments to be displayed to two expert clinicians, who marked the locations of contractions on each trace. The traces were only subject to a moving average filter, and no attempt was made by the study device to algorithmically differentiate between actual contractions and potentially misleading features such as movement of the child or mother. The axis limits corresponding to tocodynamometer output used for traces collected by the commercial external tocodynamometer were set to the maximum and minimum values the device could output. The axis limits corresponding to tocodynamometer output for traces generated by the Optoco device were selected as the 5th and 95th percentiles of the signal, excluding points below a minimum signal threshold, and expanded by 10% of the total range. Clinicians were blinded to the source device of each trace, and traces were presented in a random order. The clinicians had access to the patient’s weight, cervical dilation, and time of delivery when annotating the traces.

Patients were excluded from the analysis if less than ten minutes of data were collected, or if incomplete data was collected from one or both devices. A patient was deemed to have incomplete data if measurements were not recorded for at least one device, or if the operator indicated a physical or electrical failure in one of the devices. The first ten patients were excluded as part of a planned training set for learning to place and operate the devices. Trace pairs were excluded from the analysis if, over any 3 minute segment, the signal was saturated or out of the selected limits for 2 or more minutes.

To evaluate the concordance between the two devices, we use the Contraction Consistency Index (CCI) defined by Jezewski et. al. [15]:


$$CCI\;=\;\frac{N_{C}}{\frac{1}{2}(N_{T}+N_{E})}$$ (1)

Where N_T_ is the number of contractions marked on the Optoco device, N_E_ is the number of contractions marked on the commercial external tocodynamometer, and N_C_ is the number of consistent contractions between the two. A consistent contraction was defined as one where the peaks marked on each device were within 30 seconds of each other [16]. A similar consistency metric was used to evaluate interobserver agreement, with N_E_ and N_T_ replaced with the total number of contractions annotated by either observer. The same 30 second window was used to define consistency of an individual contraction. The trace segments were also categorized according to the frequency of annotated contractions. Traces were split into 3 categories based on the number of contractions per segment: tachysystole (5 or more), adequate (2–4), and inadequate (0–1).

## Results

Sixty patients were enrolled in the study. Of these, five (8.3%) were excluded because no data was collected on either device due to the patient delivering or revoking consent before data collection commenced. Seven (11.6%) were excluded because incomplete or insufficient data was collected from one or both of the devices. Of these seven, one was excluded because fewer than 10 minutes of data were collected, four were excluded due to a hardware failure in the study device, one was excluded due to a failure in the Corometrics device, and one was excluded due to a software issue. The first ten (16.6%) patients were excluded as a training set for learning to place and operate the devices. From the remaining 38 (63.3%) patients, there were 342 pairs of 10 minute traces, with an average monitoring duration of 92 minutes per patient. Table 1 shows a breakdown of demographic information collected about the patients whose traces were used in the analysis. Using the quality control metric described in the analysis section, 25 of these pairs were excluded from the analysis.

**Table 1:** Study demographics for analyzed patients.

	Mean	Median	Standard Deviation
Maternal Age (years)	25.00	25.5	5.30
Gravidity	2.16	2	1.46
Parity	1.05	0	1.47
Gestational Age (weeks - days; rounded to the nearest day)	38 - 0	38 - 0	2–2
Maternal Weight (kg)^†^	65.97	64.3	9.80
Cervical Exam (cm)^††^ Latent Labor (0–5 cm, %) Active Labor (6–10 cm, %)	4.89 66% 29%	5	1.76

†Weight data missing from one patient

††Cervical Exam data missing from two patients

**Fig 2:**
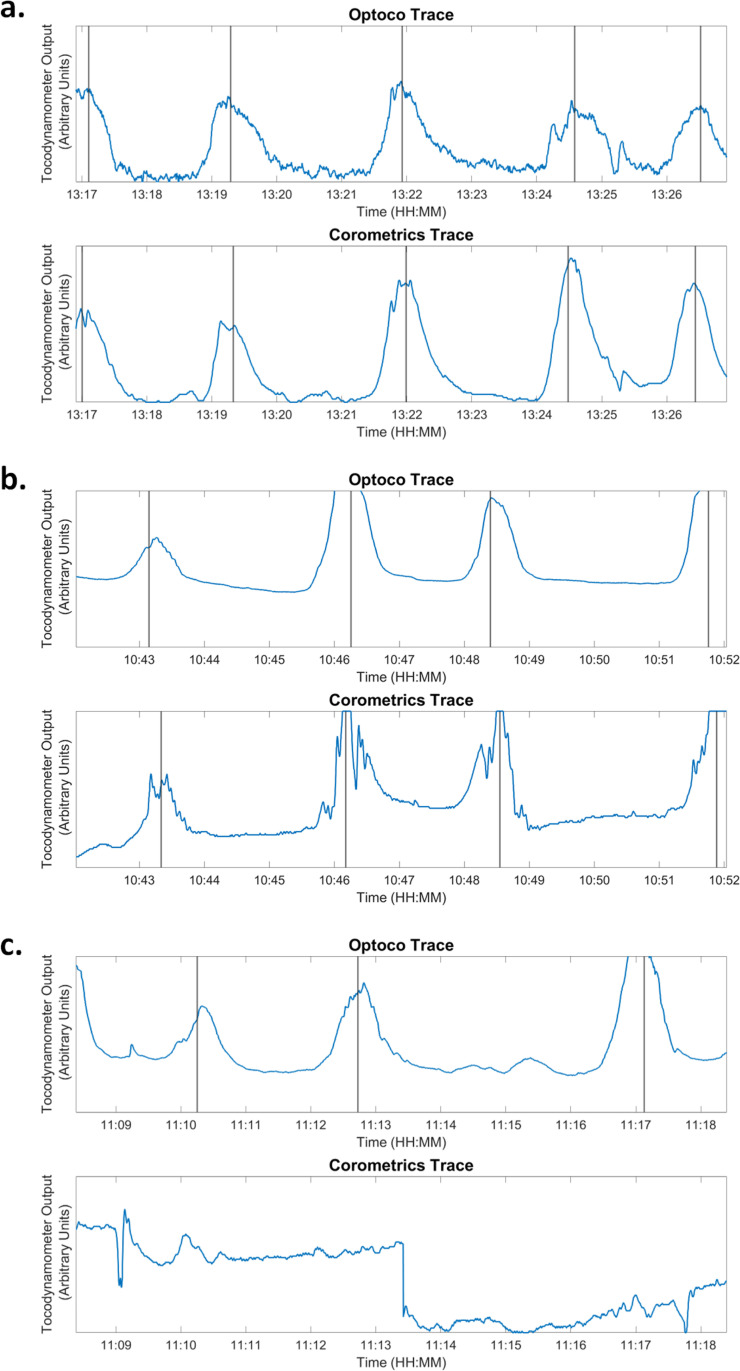
Annotated traces. Comparisons of annotated contemporaneous traces from the Optoco device and the Corometrics Toco with a Contraction Consistency Index of 1 **(a and b)**, and a Contraction Consistency Index of 0 **(c)**.

Up to two trace pairs were selected randomly from each patient for annotation by expert clinicians. One patient had only one trace pair to select. In total, 75 trace pairs were annotated. Fig 2 shows three examples of annotated contemporaneous traces from each monitor. The trace pairs shown in a. and b. display excellent agreement, with per trace CCI values of 1. In the trace pairs shown in c., several contractions are annotated on the Optoco trace, but none are noted on the Corometrics trace. This could be due to improper placement of the Corometrics tocodynamometer.

For each observer, Table 2 summarizes the number of contractions annotated for the 75 contemporaneous segments from the two devices, along with the number of consistent contractions between the two devices. Observer 2 annotated 14% more contractions on traces from the Optoco device, and 26% more contractions on traces from the Corometrics tocodynamometer when compared to observer 1. However, the CCI values for the two observers were similar. Table 2 also summarizes the agreement between the two observers.

**Table 2:** Summary of all contractions annotated by the clinicians on both devices.

	Total Number of Optoco Contractions Annotated	Total Number of Corometrics Contractions Annotated	Total Number of Consistent Contractions Between Devices	Overall CCI [95% Confidence Interval]
Observer 1	226	206	141	0.65 [0.58 - 0.72]
Observer 2	258	259	171	0.66 [0.60 - 0.72]
Number of Consistent Contractions Between Observers	216	195		
Consistency Index [95% Confidence Interval]	0.89 [0.84 - 0.93]	0.84 [0.78 - 0.88]		

**Fig 3:**
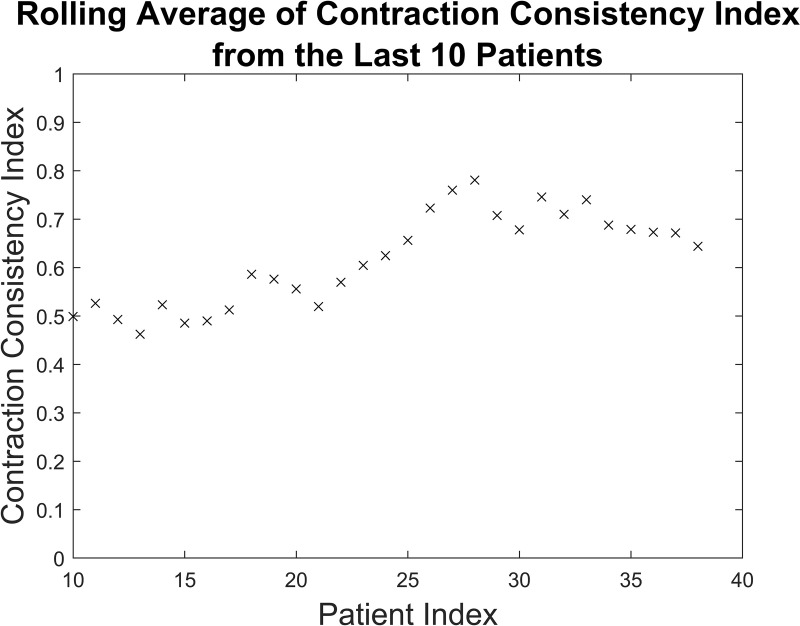
Average contraction consistency index over time. Rolling average of the contraction consistency index from the last ten patients for both observers.

An analysis was performed to examine the changes in CCI values over the duration of the study. We computed the rolling average of the CCI for the two observers for contemporaneous traces in 10 patient batches; Fig 3 shows the average CCI value vs the last patient included in the average (range patient 10–38). The average CCI value for each ten patient batch increased from 0.50 to 0.64 over the course of the study, with a peak value of 0.78, although these changes were not statistically significant (p = 0.272).

The annotated traces were also analyzed to determine whether any differences in clinical management would have resulted from the two devices. Traces were classified into three groups based on the number of contractions annotated in each segment: tachysystole (5 or more), adequate (2–4), and inadequate (0–1). Table 3 shows a breakdown of the traces into these categories and the agreement between the two devices. There was perfect agreement between the annotated results in 72% of cases. Annotated traces from Optoco were more likely to show tachysystole than those recorded with the Corometrics monitor.

**Table 3:** Consistency of clinical recommendations by device (both observers).

		Optoco		
		Inadequate	Adequate	Tachysystole
Corometrics	Inadequate	9 (6.0%)	13 (8.7%)	0 (0.0%)
	Adequate	7 (4.7%)	83 (55.3%)	14 (9.3%)
	Tachysystole	1 (0.7%)	7 (4.7%)	16 (10.7%)

## Discussion

Proof-of-concept study results comparing the Optoco device to a commercially available external tocodynamometer suggest reasonable agreement between the two. The overall CCI for both observers was 0.66, with the average CCI increasing from 0.50 for the first 10 patients to 0.64 for the last 10 patients. This could be related to the importance of proper positioning of external tocodynamometers in getting an accurate signal [17]. The nurses placing the devices had limited experience with external tocodynamometers, and their increased familiarity with the devices likely led to better placement of them as the study progressed.

The consistency between the two observers in marking contractions when compared against each other was 0.87 for both devices, using the CCI metric. This likely represents an upper bound on the agreement that could be expected from a subjective comparison of the traces. The interobserver consistency is slightly higher for the Optoco device than the external tocodynamometer (0.89 vs 0.84).

For the seventy-five contemporaneous trace pairs analyzed, the two devices provided the same clinical recommendation 72% of the time on a per trace basis. In cases where clinical recommendations differed, the Optoco device was more likely to overestimate the number of contractions when compared to the Corometrics tocodynamometer. The reasons for this difference are unclear, and would require further experimentation to investigate.

Existing external tocodynamometers can cost several hundred dollars, with the additional cost of the fetal monitoring device and software needed to use them approaching several thousand dollars. This can be prohibitively expensive in low resource settings, where low nurse to patient ratios have a significant impact on the ability to manually conduct fetal and maternal monitoring. Other emerging devices based on electrohysterography are designed to measure multiple types of signals simultaneously, and with the exception of the ANNE, interface with their own proprietary display and recording devices, increasing their cost. The Optoco device’s status as single purpose hardware could make integrating it into existing hospital or clinic workflows simpler and less expensive. Optoco could help extend access to continuous uterine activity monitoring to these areas, which may help avoid adverse neonatal outcomes related to uteroplacental insufficiency secondary to uterine tachysystole [18]. Furthermore, the Optoco device was tested in a low resource setting where the device is intended for use, suggesting generalizability to other low resource settings. Additionally, although training was provided prior to study commencement, the nurses who placed the devices did not routinely use external tocodynamometery. The temporal improvement in the agreement between the two devices suggests that results could improve with more comprehensive training or improvements to the usability of the device.

The primary limitation of this study is that the reference standard, commercial tocodynamometry, is subject to the same problems with signal acquisition due to placement on the abdomen as the study device, Optoco. Furthermore, because optimal placement of the reference standard is affected by the placement of the study device, the accuracy of the reference standard may suffer as a result, complicating interpretation of the results. Future studies should use an IUPC as the gold standard to better evaluate the accuracy of the device, though this could be difficult given that IUPCs are often unavailable in low resource settings, and in high resource settings are generally reserved for situations where accurate readings from an external tocodynamometer cannot be obtained due to the risk of complications [19,20]. Both devices also do not provide information about contraction magnitude. The subjective nature of uterine contraction trace interpretation can also complicate comparisons of the two devices, with improper positioning of the devices, high patient BMI, or artifacts caused by movement of the mother or child resulting in potential under or overcounting of contractions. Another limitation of the study was the prevalence of patients with insufficient or incomplete data (11.6%). Improvements to the reliability of electronics components in the device could help reduce this number in future studies. The relatively small sample size and high overall patient level exclusion rate (36.7%) also limit the generalizability of the results. Furthermore, as this was a proof of concept pilot study, a formal power calculation was not completed prior to commencement of the study. The device’s performance was not tested on pre-labor contractions, but we have no reason to believe that it would be significantly different.

Future studies of the Optoco device using an IUPC as the gold standard could allow for a more effective characterization of the device’s accuracy, while future studies comparing the device to uterine electromyogram devices could improve our understanding of the device’s performance relative to existing alternatives. The device could also be tested in concert with other existing or developing fetal monitoring technologies to potentially increase its clinical impact. The annotation data collected in this proof-of-concept work here could be used to produce an algorithm designed to count contractions in future works. This information could then be used to develop a comprehensive plan to evaluate the potential for automated contraction detection, integration with fetal heart rate monitoring, and broader usability testing of the device, particularly in settings where external tocodynamometers are not routinely available.

### Conclusions

The low cost external tocodynamometer presented here provides reasonable agreement with an existing commercial external tocodynamometer. Due to limitations in the study design regarding placement of the two devices and the small sample size, further evaluation of the device’s usability and accuracy in future studies are needed, as are the clinical benefits to the maternal-neonatal dyad.

## Supporting information

S1 Fig Data flow diagram. An outline of the processing and analysis steps applied to the data from each device.
(TIF)

S1 Checklist Questionnaire on inclusivity in global research. (DOCX)
